# Intrinsic Variability in Shell and Soft Tissue Growth of the Freshwater Mussel *Lampsilis siliquoidea*

**DOI:** 10.1371/journal.pone.0112252

**Published:** 2014-11-20

**Authors:** James H. Larson, Nathan L. Eckert, Michelle R. Bartsch

**Affiliations:** 1 U.S. Geological Survey, Upper Midwest Environmental Sciences Center, La Crosse, Wisconsin, United States of America; 2 U.S. Fish and Wildlife Service, Genoa National Fish Hatchery, Genoa, Wisconsin, United States of America; Gettysburg College, United States of America

## Abstract

Freshwater mussels are ecologically and economically important members of many aquatic ecosystems, but are globally among the most imperiled taxa. Propagation techniques for mussels have been developed and used to boost declining and restore extirpated populations. Here we use a cohort of propagated mussels to estimate the intrinsic variability in size and growth rate of *Lampsilis siliquoidea* (a commonly propagated species). Understanding the magnitude and pattern of variation in data is critical to determining whether effects observed in nature or experimental treatments are likely to be important. The coefficient of variation (CV) of *L. siliquoidea* soft tissues (6.0%) was less than the CV of linear shell dimensions (25.1–66.9%). Size-weight relationships were best when mussel width (the maximum left-right dimension with both valves appressed) was used as a predictor, but 95% credible intervals on these predictions for soft tissues were ∼145 mg wide (about 50% of the mean soft tissue mass). Mussels in this study were treated identically, raised from a single cohort and yet variation in soft tissue mass at a particular size class (as determined by shell dimensions) was still high. High variability in mussel size is often acknowledged, but seldom discussed in the context of mussel conservation. High variability will influence the survival of stocked juvenile cohorts, may affect the ability to experimentally detect sublethal stressors and may lead to incongruities between the effects that mussels have on structure (via hard shells) and biogeochemical cycles (via soft tissue metabolism). Given their imperiled status and longevity, there is often reluctance to destructively sample unionid mussel soft tissues even in metabolic studies (e.g., studies of nutrient cycling). High intrinsic variability suggests that using shell dimensions (particularly shell length) as a response variable in studies of sublethal stressors or metabolic processes will make confident identifications of smaller effect sizes difficult.

## Introduction

Bivalves are ecologically and economically important members of many aquatic ecosystems, providing physical structure [1], influencing biogeochemical cycles [2], [3] and influencing primary and secondary production [4], [5]. Within North American waters, most native mussels fall within the family Unionidae (superfamily Unioniformes; [6], [7]) and these native mussel species are considered among the taxa most susceptible to extinction in the near-future [8]. For this reason, management of native unionid mussel populations has been a focus of natural resource agencies in both the United States and Canada [9].

Propagation and subsequent release of these hatchery-raised mussels for reintroduction to areas where they have been extirpated (or to augment low existing populations) has become a widely-used strategy in North America and elsewhere [9], [10]. Controlled propagation involves infecting fish hosts with glochidia (the parasitic larval stage of unionid mussels) and either releasing those fish hosts immediately back into the wild or holding them within field or hatchery enclosures until individual mussels fall off the fish (the juvenile stage). Juveniles are then stocked prior to reaching reproductive age.

A cohort of juveniles produced via controlled propagation has been treated similarly throughout their life. As a result, these artificially reared cohorts can be used to estimate variability in mussel size (shell dimensions), growth and mass (tissue dry weight) independent of the many differences that would drive variation in body morphology in wild populations (e.g., temperature, habitat suitability, food availability). Understanding the magnitude and pattern of variation in data is critical to determining whether effects observed in nature or experimental treatments are likely to be important. For example, if mean growth rate declines by 5% due to ambient concentrations of a stressor, but underlying variation in the mean growth rate is very high (coefficient of variation >50%), then the effect of the stressor, though real, will be difficult to detect in natural populations.

Here, we measured the size and mass of a single cohort of artificially propagated mussels. The primary objective was to understand the magnitude and structure (i.e., distribution) of variation in size (shell dimensions), mass (dry weight) and interactions between these measurements (the accuracy of length-weight regressions) in *Lampsilis siliquoidea* (fatmucket).

## Methods

### Ethics statement

A Wisconsin Department of Natural Resources scientific collection permit issued to NLE covers the mussel collection described herein. No endangered or protected species were involved in this study.

### Propagation methods

Gravid female *L. siliquoidea* were collected from the St. Croix River (near Houlton, WI; ∼45.0559°N, −92.8008°E) in April 2011 and used to inoculate Largemouth Bass, *Micropterus salmoides*, at Genoa National Fish Hatchery (NFH). Three gravid females were used to inoculate fish. Glochidia were extracted via the syringe method and fish were inoculated in a glochidia bath (specific inoculation rates were not measured). The inoculated host fish were placed in several mussel culture cages and were subsequently placed in the St. Croix River. Mussel culture cages are 0.91 by 0.61 by 0.46 m in size (3×2×1.5 feet). Based on temperature data collected at a nearby location in the St. Croix River (USGS gage 05340500), mean daily water temperatures ranged from 1.7–29°C during the time period the mussels were in the river. In September 2012, the cages were retrieved from the St. Croix River and the resulting juvenile mussels were transported to the Genoa NFH where they were held in a raceway (4.3×9.1 m; ∼7 individuals per m^2^) supplied with pond water at an ambient temperature until the onset of the present study. All of the juvenile mussels were retrieved from the raceway in May 2013 (275 total) at which time shell dimensions and mass were measured.

### Mussel measurements

Several physical properties of the mussels were measured. Length, width and height were all recorded using a digital caliper that reported to the 0.01 mm. Length here is the maximum anterior to posterior dimension of the shell measured roughly parallel to hinge, width is the maximum left-right dimension with both valves appressed and height is the maximum dorsal-ventral dimension of the shell measured roughly perpendicular to the hinge. On a separate set of 12 mussels (36 measurements), measurement error by JHL (who conducted most of the measurements here) was estimated to be less than 0.2% on average (range 0–2.3%; see Appendix S1). Two-hundred and twenty five mussels were then randomly selected to be used in another study. The remaining 50 mussels were analyzed for dry mass and ash-free dry mass (AFDM). These mussels were initially frozen, and then the soft tissues were removed and shells and soft tissues were placed into separate, pre-weighed tins for drying at 60°C (>24 h). After drying, individual tins were weighed, and then placed into a muffle furnace and ashed (550°C for1 hr) before being weighed a final time. Ash-free dry mass (AFDM) was calculated by subtracting the ashed mass from the dry mass.

### Statistical analysis

All statistical analyses were performed in R [11]. Average, standard deviation(SD), coefficient of variation (CV) and 95% credible intervals were estimated using a Bayesian approach described by McCarthy [12]. Based on visual inspection of the data, three possible distributions were considered for the physical data: Normal, log-normal and gamma. These were compared using the BRugs package in R [13], which connects R to OpenBUGS [14].

Simple linear models (M =  *b*D+*a*; where M is the mass and D is the shell dimension) and logarithmic models (M =  *a*D*^b^*; [15]) relating physical dimensions to mass were compared by estimating model parameters, Bayesian correlation coefficients (R^2^_B_; [16]) and the Deviance Information Criterion (DIC, [12]). DIC is similar to the more widely used Akaike Information Criterion (AIC; [17]). A DIC value is generated for each model, and the model with the lowest DIC is considered to fit the data best. The actual DIC value is not meaningful, so it is the comparison of a particular model to the best model (the ΔDIC) that is most useful. Models with a ΔDIC <2.0 are generally considered 'strongly supported'. Code and additional statistical details are included in Appendix S2. Raw data is provided in Appendix S1.

A hypothetical example is provided to illustrate the effects of estimated variation on the ability to detect differences among populations experiencing different levels of a stressor. A sample of 5, 10 or 20 individual observations were derived from normal distributions representing the growth of the mussels measured in this study, and the growth of mussels hypothetically experiencing a stressor that reduced the mean and standard deviation of growth by 5, 10, 25 and 50%. Credible intervals (95%) were then constructed using those derived ‘observations’ to estimate whether differences between these different effect sizes would be readily detectable at the different levels of sampling effort.

## Results

Soft tissues were separated from the shell prior to weighing mussels, but these soft-tissues accounted for only 60% (range 46.0–69.6%) of the total mussel organic material (as indicated by ash-free dry mass [AFDM]). The remaining organic material occurred in the shells, although this was a small percentage of the total shell mass (shell AFDM 5.9% of shell dry mass [DM]; standard deviation 0.24%). These two sources of mussel organic matter were tightly correlated (shell and soft tissue AFDM Pearson's *r* = 0.95), so total mussel organic matter is used in the following analysis (shell plus soft tissue AFDM).

**Figure 1:**
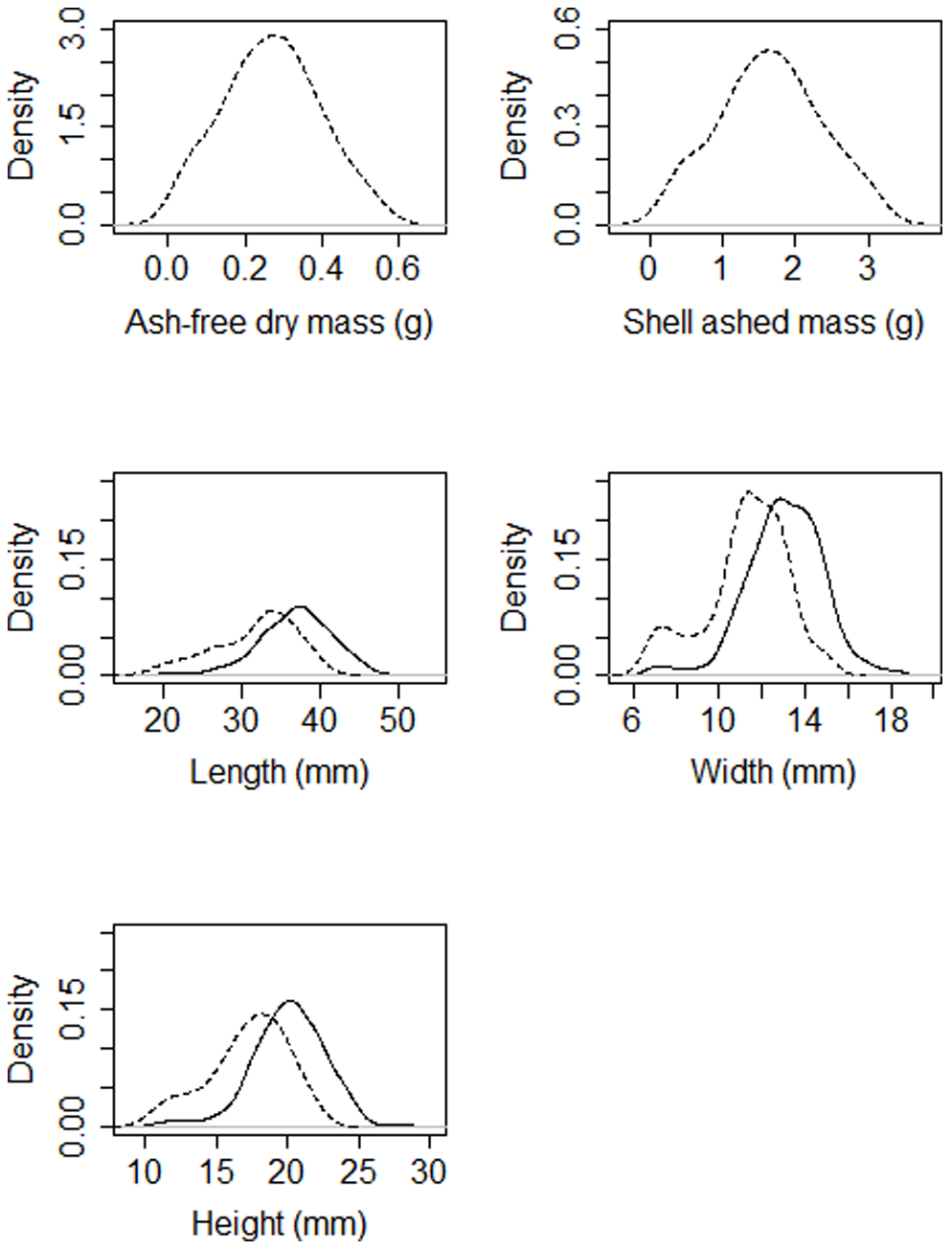
Density distributions of body characteristics derived using kernel density estimation. Dashed lines indicate distributions derived using only the 50 individuals sampled for dry mass. Solid lines are distributions derived using all 275 individuals sampled. Note that shell dimensions (width, length and height) in the sub-sample of mussels sampled for dry mass tend to overrepresent the smallest individuals. Normal distributions were good approximations for all of these data (Table 2).

Total organic matter (AFDM) in the 50 mussels measured here was considerably less variable (CV 6.0%) than the mass of the shells (shell DM minus shell AFDM  =  shell ashed mass; CV 31.2%) or the individual shell dimensions (CV length  = 66.9%, CV width  = 25.1%, CV height  = 36.3%; Table 1). Ratios between shell dimensions (length, width and height) were much less variable (CV ≤1.0%; Table 1). A normal distribution was the best fit for explaining the distribution of all the measured variables (Table 2, Figure 1). The 50 mussels sampled for dry mass measurements had a distribution of shell dimensions that largely overlapped with the distribution of the entire 275 mussel cohort, shifted slightly to the smaller sizes (Figure 1).

**Table 1:** Size and mass of 275 *Lampsilis siliquoidea* (fatmucket) mussels artificially propagated and reared to age 2.

Dimension	Mean (SD)	CV	Range (min-max)	95% credible interval*	*n*
Ash-free dry mass (mg)	269.4 (16.1)	6.0%	45.9–541	233.8–304.8	50
Ash-free dry mass -soft tissue only (mg)	168.2 (8.1)	4.8%	22.9–372.5	143.0–193.2	50
Shell ashed mass (mg)	1,654 (513)	31.2%	389–3,050	1,453–1,853	50
Shell length (mm)	36.8 (24.6)	66.9%	19.5–50.5	36.2–37.4	275
Shell width (mm)	13.1 (3.3)	25.1%	6.91–18.3	12.9–13.3	275
Shell height (mm)	20.0 (7.26)	36.3%	9.92–28.1	19.7–20.4	275
Length:Width	2.82 (0.29)	1.0%	1.9–3.25	2.80–2.84	275
Width:Height	0.654 (0.006)	0.9%	0.55–1.75	0.646–0.663	275
Length:Height	1.84 (0.014)	0.8%	1.53–3.33	1.826–1.854	275

CV- coefficient of variation; SD - standard deviation.

*95% credible intervals are based on a normal distribution for all variables.

**Table 2:** Relative fit (ΔDIC) of normal, gamma and log normal distributions to the mussel characteristics in artificially reared *Lampsilis siliquoidea*.

Response variable	Normal	Gamma	Log Normal
Ash-free dry mass	**0**	4.1	12.8
Shell ashed mass	**0**	3.9	11.1
Shell length	**0**	21	35
Shell width	**0**	19	33
Shell height	**0**	23	40
Length:Width	**0**	22.7	40.9
Width:Height	**0**	**0**	26
Length:Height	**0**	5.5	69.4

Fifty individuals were used for dry mass measurements and 275 individuals were used for shell dimensions.

**Figure 2:**
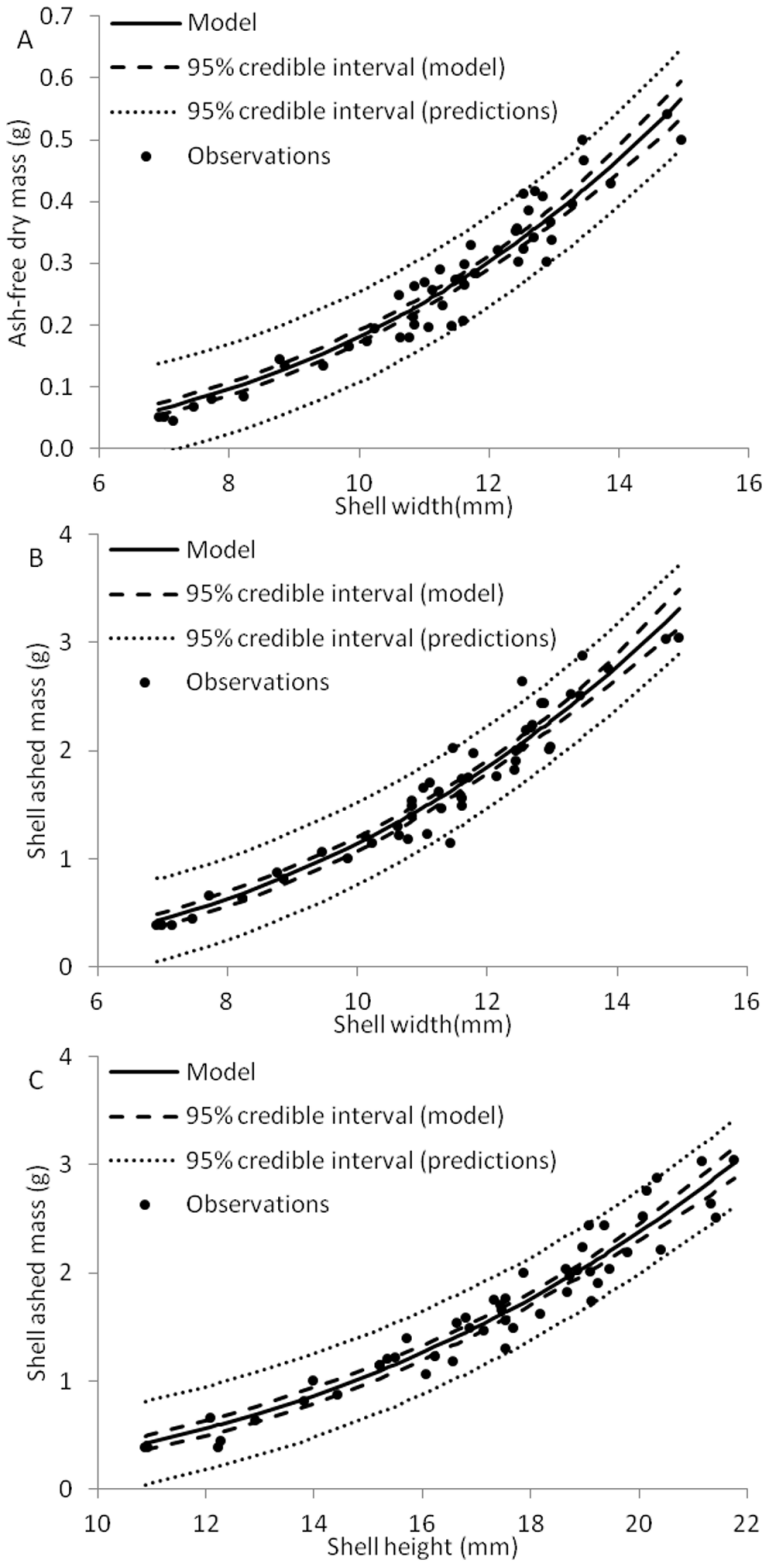
Models relating shell dimensions to ash-free dry mass (A) and shell ash-free dry mass (B,C) in juvenile *Lampsilis siliquoidea*. The model line shows the average mass for the population at a particular shell width (or height), the model 95% credible interval shows the interval for the population at that shell width, and the prediction 95% credible interval shows the range in which 95% of the mussels sampled at that width (or height) are likely to occur. Model details are in Table 3. Only strongly supported models are depicted here (ΔDIC <2.0).

Relationships between shell dimensions and mussel mass were very strong, with Bayesian R^2^_B_ values of 0.91 for organic matter (AFDM) and 0.93 for shell mass (ashed mass) in the best model (Table 3, Figure 2). Logarithmic model forms were always better than the equivalent linear model (as determined by ΔDIC) and the best models for organic matter and shell mass both included width as the best predictor (Table 3). A logarithmic model with shell height as a predictor of shell mass was equally supported by the data (ΔDIC of 1.06; Table 3). Mean values of *b* in the logarithmic models were always below 3 in strongly supported models (indicating negative allometry), but 95% credible intervals overlapped 3 for the model relating organic matter to shell width and the model relating shell mass to height (Table 3). Even with these high R^2^_B_ values, 95% credible intervals on predictions were often wide. For example, the 95% credible interval on a prediction of organic matter (AFDM) using the best model is still ∼145 mg wide, which is about half the mass of an ‘average’ individual (Table 1, Figure 2).

**Table 3:** Relationships between shell dimensions and tissue dry mass for 50 artificially propagated juvenile *Lampsilis siliquoidea* mussels.

Dry Mass	Model*	*b* ‡	*a* ‡	R^2^_B_ ‡	ΔDIC
Ash-free dry mass	**Logarithmic W** †	**2.87_[2.58 to 3.16]_**	**0.18_[0.17 to 0.19]_**	**0.91_[0.87 to 0.94]_**	**0**
	Linear W	0.61_[0.55 to 0.67]_	−0.42_[−0.48 to–0.35]_	0.89_[0.83 to 0.93]_	11.9
	Logarithmic H	3.05_[2.67 to 3.44]_	0.05_[0.04 to 0.06]_	0.88_[0.83 to 0.92]_	14.8
	Logarithmic L	3.07_[2.65 to 3.53]_	0.01_[0 to 0.01]_	0.87_[0.8 to 0.91]_	17.7
	Linear H	0.42_[0.38 to 0.46]_	−0.45_[−0.53 to_ –_0.38]_	0.86_[0.8 to 0.91]_	22.3
	Linear L	0.22_[0.2 to 0.25]_	−0.43_[−0.51 to_ –_0.35]_	0.84_[0.77 to 0.9]_	29.1
Shell ashed mass	**Logarithmic W** †	**2.64_[2.39 to 2.89]_**	**1.14_[1.07 to 1.22]_**	**0.93_[0.89 to 0.95]_**	**0**
	**Logarithmic H** †	**2.83_[2.56 to 3.11]_**	**0.34_[0.280 to 0.40]_**	**0.92_[0.89 to 0.95]_**	**1.06**
	Linear H	2.42_[2.21 to 2.63]_	−2.51_[−2.87 to_ –_2.14]_	0.91_[0.86 to 0.94]_	12.581
	Linear W	3.46_[3.15 to 3.76]_	−2.24_[−2.58 to_ –_1.89]_	0.90_[0.856 to 0.936_]	13.508
	Logarithmic L	2.78_[2.42 to 3.14]_	0.07_[0.04 to 0.10]_	0.88_[0.82 to 0.92]_	23.582
	Linear L	1.25_[1.11 to 1.39]_	−2.31_[−2.74 to_ –_1.86]_	0.86_[0.79 to 0.90]_	33.31

*Linear models are of the form M =  *b*D+*a*; logarithmic models are of the form M = *a*D*^b^*; where M is the mass in g and D is the shell dimension (length [L], width [W] or height [H]) in cm.

† Models highlighted with **bold** are strongly supported by the DIC model selection procedure.

‡ Model parameters are indicated with 95% credible intervals in [brackets].

**Figure 3:**
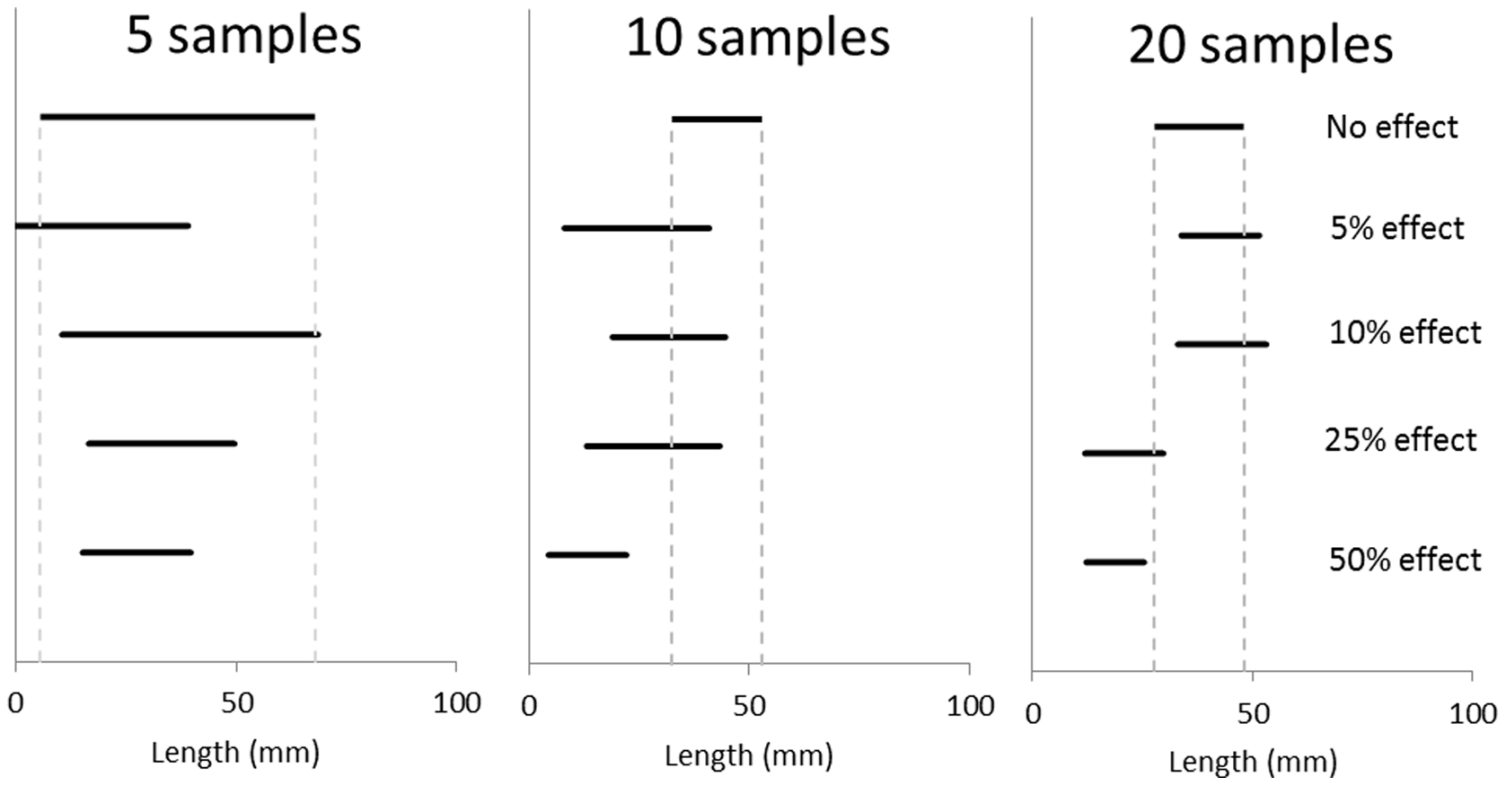
Example of how sample size and a stressor might influence estimates of mussel size in *Lampsilis siliquoidea.* Horizontal bars indicate the 95% credible interval of estimates of mean mussel size derived from either 5, 10 or 20 samples drawn from a normal distribution representing the samples collected in this study (no effect), or hypothetical samples collected from mussels where a stressor had reduced size by 5, 10, 25 or 50%. Vertical lines highlight the 95% credible interval for the “No effect” sample.

As a thought experiment, we considered how high variability in shell length might influence the ability to detect the effects of a stressor on mussel growth. In this hypothetical example, several groups of mussels have been raised under the same conditions as the mussels sampled here, but with different levels of a stressor. When the number of samples per treatment is low (5), even 50% reductions in average mussel size do not appear different than the no stressor control (using 95% credible intervals; Figure 3). Even when the sample number is increased four-fold, smaller effect sizes (5 or 10% decreases in size) do not have effects that are readily apparent (Figure 3).

## Discussion

The individuals sampled here were from the same cohort and reared under the same conditions. As a result, differences in size may be caused by differences in juvenile growth rates, initial size of juveniles following transformation or even initial glochidial size. High variability in growth or size (>50% CV) is common in non-unionid bivalves (e.g., in oysters, [18] and *Phacosoma japonicum*, [19]) and differences among individuals in growth rate are often attributed to genetic variation [20] although this does not seem to have been documented for unionids. Within unionids, high variability is also evident in early life history stages [21] and within propagation systems (B. Simmons, pers. comm.). Some toxicity studies report much lower variability in growth or size than observed here. For example, *L. siliquoidea* populations in a study on copper (Cu) toxicity had much less variability in size after a 28-day growing period [22]. Wang et al. [22] looked at the survival and size (shell length) of *L. siliquoidea* raised without Cu in several food conditions and found within-treatment CVs ranged from 8–22%, much lower than those observed here. The data presented here provides little insight into the potential mechanism causing high variability. Genetic variation, high infestation density on host fish, microhabitat differences or subtle differences in the transformation from glochidia to juvenile stages could all play a role in creating the variation in size and apparent growth rate documented here.

Shell dimensions were related to the shell mass with a logarithmic relationship reminiscent of typical cube-law relationships used in studies of fish and other bivalves [15], [23], with a tendency towards negative allometry (*b*<3). However, unlike most other studies relating linear dimensions to mass, the individuals sampled here do not represent a continuum of ages but a single cohort growing at different rates [15], [23]. Negative allometry in this context is somewhat more difficult to interpret, but it appears that faster growing individuals are proportionally wider than slower growing individuals, at least in terms of shell mass (note that the logarithmic model fitted here uses width not length as is commonly the case when interpreting allometry; [15]).

Although acknowledged informally, the implications of high variability in growth do not appear to have been often discussed in the context of conservation or ecology of these species [10]. The high variability in shell growth and size, even when conditions are nearly identical, has at least three important implications for the ecology and conservation of mussels.

### Juvenile survival to adulthood

Shell size and survival are thought to be correlated in mussels [24]–[27]. For species of conservation concern, these size-related differences in survival could have a significant impact on the success of stocking programs. In the models of Villella et al. [25], survival of *L. cariosa* increased nearly linearly between 20–50 mm shell length, and then increased only slightly between 60–100 mm. This suggests that once past some critical size range, high survival is likely. The size at which 80% survival could be expected differed among species (∼50 mm in *L. cariosa*, ∼35 mm in *Elliptio complanata*, ∼70 mm in *E. fisheriana*), indicating significant among-species variability [25]. *L. siliquoidea* are quite different in terms of shell shape and size at adulthood than the species studied in Villella et al. [25], but it is likely that some similar relationship exists connecting size to survival in *L. siliquoidea.* Within the cohort studied here, differences likely exist among individuals in the likelihood of survival to reproductive age had they been stocked. Establishing a size-survival relationship would allow for the creation of size-at-stocking thresholds for propagated mussels, but this seems to be unknown for most species.

### Small-scale drivers of mussel ecology

Variation in the size of the *L. siliquoidea* measured here is reflective of differences in growth or development rate, since all individuals infected fish at approximately the same time. This is intrinsic variation (presumably genetic; [20]) that occurs even when habitat conditions are similar. When intrinsic variation is high, external drivers of mussel growth must have large effect sizes to have an effect on mussel size that could be easily documented *in-situ* or in long-term studies. In our hypothetical stressor example, small samples sizes would probably be incapable of establishing whether stressor effects were even significantly different than zero, let alone estimate the magnitude of the effect. Even with very large sample sizes, if a stressor causes an effect on size that is less than the coefficient of variation, then the mussels influenced by the stressor will still have a shell length that is within 1 standard deviation of mussels grown without the toxin. Considering the high CVs observed here for commonly measured shell dimensions (66.9% for shell length), this implies only large effects may be detectable even when samples sizes are quite high. For studies attempting to document the impacts of stressors or other environmental parameters on growth in field studies, these issues will be compounded by differences in cohorts and antecedent conditions.

### Disconnects between structural and metabolic impacts of mussels

Mussels influence aquatic ecosystems in a variety of ways; many of these influences can be described as structural or metabolic. Structural influences include the ability of mussels to provide substrate for other species [28], whereas metabolic processes include the direct role of mussels on food web structure and nutrient cycling [5], [29]. There is general reluctance to destructively sample unionid mussels due to their longevity and imperiled status, and therefore researchers use shell dimensions (often shell length) as a surrogate for soft-tissue mass to minimize the need to destructively sample (e.g., [30]). In this study, while variability was high for shell dimensions (particularly shell length, 66.9%), the variability in organic matter was much lower (only 6.0% for total AFDM). Clearly the dynamics of shell and soft-tissue growth differ to some degree in this species. Although shell size was strongly related to soft-tissue biomass in this cohort, even the best model still suggests multiple mussels at a similar shell size could have quite different soft-tissue biomass. For example, we observed four individuals with shell widths that varied by less than a tenth of a mm (11.58–11.61 mm) that had quite different organic matter content (AFDM  = 207–300 mg, a 45% increase from the smallest individual to the largest). None of these individuals fell outside the 95% credible intervals for predictions from the best model relating linear shell dimensions to AFDM. Presumably, variation in metabolism in mussels would be more closely tied to variation in organic matter (i.e., AFDM) than hard tissue dimensions. In natural systems, where a particular size class would likely be composed of many individual cohorts exposed to variation in environmental conditions, the variability in soft-tissue mass within a size class may be even greater. For *L. siliquoidea* in particular, shell length did not appear to be as good an indicator of soft tissue mass as other shell dimensions (width).

## Conclusions

Unionid mussels are among the most imperiled taxa in North America [10], as a result they are often the focal point of regulatory actions and conservation efforts (e.g., [31]). Understanding the controls over the survival of unionid mussels is a critical need if they are to be restored to their former abundance [32]. Size appears to be one important control over survival [24], [25], but the analysis here suggests size will vary substantially even when environmental conditions are similar. This variability will make it difficult to identify sub-lethal drivers of mussel growth or size in natural settings.

This study focused on a single cohort of *L. siliquoidea*, but conversations with other propagation experts, our personal experience and publications on earlier life-history stages suggests this level of variability is not uncommon in many mussel species (B. Simmons, pers. comm.; [18], [21]). For the purpose of stocking mussels, this may imply that stocking entire cohorts will have a lower probability of success than stocking only individuals that have attained a particular size threshold. Establishing the size threshold that achieves conservation goals will probably require *in-situ* mark-recapture studies similar to those done by Villella et al. [25], perhaps using propagated mussels as test subjects.

## Supporting Information

Appendix S1 **Data associated with this manuscript.** Spreadsheets in this file contain linear shell dimensions, tissue masses, data to estimate precision and a meta-data page that identifies the column labels.
(XLSX)

Appendix S2 **Example code for the regression analysis used in this paper.**
(DOCX)
