# Supplementary material for: Intrinsic Variability in Shell and Soft Tissue Growth of the Freshwater Mussel Lampsilis siliquoidea
Source: PLoS One. 2014 Nov 20;9(11):e112252. doi: 10.1371/journal.pone.0112252 (PMC4239029; doi:10.1371/journal.pone.0112252)
Supplement: Appendix S2 — Example code for the regression analysis used in this paper. (DOCX) [file pone.0112252.s002.docx]

Statistical Appendix.

Statistical analysis was conducted in R version 2.11.1 [1], using the BRugs package, version 0.7-5 [2]. Essentially, this allows R to interface with the OpenBUGS program to run Markov chain Monte Carlo simulations of models [3,4]. A detailed example of the code used for the model selection procedure is provided below. The .csv file used in this example is included as a worksheet in the Data Appendix.

Example code (with explanation) follows. In R, text following "#" symbol is not part of the code, and so explanations have been embedded in the code following the "##" symbol. File locations have been left in as examples, but for others repeating the analysis, these file locations need to be altered to the appropriate location (or the data must be imported some other way). Variable names are explained in the Data Appendix.

#################

library("BRugs") # The BRugs Package

library("plyr") #This package allows for easier sorting and ##subsetting of data.

DryMassSamples <- read.csv("~/USGS Science/Mussel Size Distribution/Analysis/DryMassSamples.csv") #The data file

DryMassSamples$AshMassTissue <-DryMassSamples$AFDM #Column mislabeled

DryMassSamples$AshMassShell <-DryMassSamples$ShellAFDM #Column mislabeled

#Calculating AFDM of soft tissues and shell tissues

DryMassSamples$AFDM <-DryMassSamples$DM-DryMassSamples$AshMassTissue

DryMassSamples$ShellAFDM <-DryMassSamples$ShellDM-DryMassSamples$AshMassShell

DryMassSamples$TotalSoftTissue<-DryMassSamples$AFDM+DryMassSamples$ShellAFDM

DryMassSamples$L<-DryMassSamples$L/10 #converting to cm

DryMassSamples$W<-DryMassSamples$W/10

DryMassSamples$H<-DryMassSamples$H/10

attach(DryMassSamples) #This allows R to see the columns as variables

##This is the linear model in OPENBugs format.

regressionmodel <- function(){

a~dnorm(0,1.0E-6)#Wide, uninformative normal distributions

b~dnorm(0,1.0E-6)

prec~dgamma(0.001,0.001)

sy2 <- pow(sd(y[]),2)

R2B <- 1 - 1/(prec*sy2)

for (i in 1:50)

{

mean [i] <- a+b*x[i]

y[i] ~dnorm(mean[i],prec)

}

}

regressionmodelfile <- file.path(tempdir(),"regressionmodel.txt")

model <- writeModel(regressionmodel,regressionmodelfile)

#This writes the model into a format OPENBugs recognizes

inits <- "C:\\Users\\jhlarson\\Desktop\\USGS Science\\OpenBugs Code\\Regression\\Initials.txt"

#Initials; list(a=0, b=0, prec=100)

x <- L

y <- AshMassShell

#defining x and y

bdata <-bugsData(c("y","x"),,digits=5) #Converts the data to a format #OPENBugs can read

modelCheck(regressionmodelfile) #Input the model

modelData(bdata) #Input the data

modelCompile(numChains=1) #Compile

modelInits(inits,) #Initialize

modelUpdate(50000) #50,000 iterations as a burn-in

samplesSet(c("b","R2B","a","prec")) #Tells R to monitor these variables

dicSet() #Tells R to monitor the DIC value

modelUpdate(50000) #50,000 iterations to run

SlopeL <- samplesStats("*")#Store data on variables

SlopeLDIC <- dicStats() # Store DIC data

SlopeL$Model<-"L" #Label this dataset with the model

SlopeLDIC$Model<-"L"

#This repeats for the other variables and models.

x <- W

y <- AshMassShell

bdata <-bugsData(c("y","x"),,digits=5)

modelCheck(regressionmodelfile)

modelData(bdata)

modelCompile(numChains=1)

modelInits(inits,)

modelUpdate(50000)

samplesSet(c("b","R2B","a","prec"))

dicSet()

modelUpdate(50000)

SlopeW <- samplesStats("*")

SlopeWDIC <- dicStats()

SlopeW$Model<-"W"

SlopeWDIC$Model<-"W"

x <- H

y <- AshMassShell

bdata <-bugsData(c("y","x"),,digits=5)

modelCheck(regressionmodelfile)

modelData(bdata)

modelCompile(numChains=1)

modelInits(inits,)

modelUpdate(50000)

samplesSet(c("b","R2B","a","prec"))

dicSet()

modelUpdate(50000)

SlopeH <- samplesStats("*")

SlopeHDIC <- dicStats()

SlopeH$Model<-"H"

SlopeHDIC$Model<-"H"

## Standardized regression between linear dimension and tissue mass

regressionmodel <- function(){

a~dgamma(0.001,0.001)

b~dnorm(0,1.0E-6)

prec~dgamma(0.001,0.001)

sy2 <- pow(sd(y[]),2)

R2B <- 1 - 1/(prec*sy2)

for (i in 1:50)

{

z[i]<- pow(x[i],b)

mean [i] <- a*z[i]

y[i] ~dnorm(mean[i],prec)

}

}

regressionmodelfile <- file.path(tempdir(),"regressionmodel.txt")

model <- writeModel(regressionmodel,regressionmodelfile)

inits <- "C:\\Users\\jhlarson\\Desktop\\USGS Science\\OpenBugs Code\\Regression\\InitialsAllo.txt"

#Initials; list(a=0.5, b=0, prec=100)

x <- L

y <- AshMassShell

bdata <-bugsData(c("y","x"),,digits=5)

modelCheck(regressionmodelfile)

modelData(bdata)

modelCompile(numChains=1)

modelInits(inits,)

modelUpdate(50000)

samplesSet(c("b","R2B","a","prec"))

dicSet()

modelUpdate(50000)

AlloL <- samplesStats("*")

AlloLDIC <- dicStats()

AlloL$Model<-"AlloL"

AlloLDIC$Model<-"AlloL"

x <- W

y <- AshMassShell

bdata <-bugsData(c("y","x"),,digits=5)

modelCheck(regressionmodelfile)

modelData(bdata)

modelCompile(numChains=1)

modelInits(inits,)

modelUpdate(50000)

samplesSet(c("b","R2B","a","prec"))

dicSet()

modelUpdate(50000)

AlloW <- samplesStats("*")

AlloWDIC <- dicStats()

AlloW$Model<-"AlloW"

AlloWDIC$Model<-"AlloW"

x <- H

y <- AshMassShell

bdata <-bugsData(c("y","x"),,digits=5)

modelCheck(regressionmodelfile)

modelData(bdata)

modelCompile(numChains=1)

modelInits(inits,)

modelUpdate(50000)

samplesSet(c("b","R2B","a","prec"))

dicSet()

modelUpdate(50000)

AlloH <- samplesStats("*")

AlloHDIC <- dicStats()

AlloH$Model<-"AlloH"

AlloHDIC$Model<-"AlloH"

SlopesAshMassShell <-rbind(SlopeL,SlopeW,SlopeH,AlloW,AlloH,AlloL)

SlopesAshMassShellDIC <-rbind(SlopeLDIC,SlopeWDIC,SlopeHDIC,AlloWDIC,AlloHDIC,AlloLDIC)

SlopesAshMassShell$MC_error<- NULL

SlopesAshMassShell$start<-NULL

SlopesAshMassShell$sample<- NULL

SlopesAshMassShell$Parameters <- SlopesAshMassShell$row.names

SlopesAshMassShellDIC<-SlopesAshMassShellDIC[c(-2,-4,-6,-8,-10,-12),]

AshMassShellResults<-join(SlopesAshMassShell,SlopesAshMassShellDIC,by="Model")

AshMassShellResults$Parameters <- SlopesAshMassShell$row.names

###########Soft Tissue AFDM################

###########Soft Tissue AFDM################

###########Soft Tissue AFDM################

###########Soft Tissue AFDM################

###########Soft Tissue AFDM################

###########Soft Tissue AFDM################

regressionmodel <- function(){

a~dnorm(0,1.0E-6)

b~dnorm(0,1.0E-6)

prec~dgamma(0.001,0.001)

sy2 <- pow(sd(y[]),2)

R2B <- 1 - 1/(prec*sy2)

for (i in 1:50)

{

mean [i] <- a+b*x[i]

y[i] ~dnorm(mean[i],prec)

}

}

regressionmodelfile <- file.path(tempdir(),"regressionmodel.txt")

model <- writeModel(regressionmodel,regressionmodelfile)

inits <- "C:\\Users\\jhlarson\\Desktop\\USGS Science\\OpenBugs Code\\Regression\\Initials.txt"

x <- L

y <- TotalSoftTissue

bdata <-bugsData(c("y","x"),,digits=5)

modelCheck(regressionmodelfile)

modelData(bdata)

modelCompile(numChains=1)

modelInits(inits,)

modelUpdate(50000)

samplesSet(c("b","R2B","a","prec"))

dicSet()

modelUpdate(50000)

SlopeL <- samplesStats("*")

SlopeLDIC <- dicStats()

SlopeL$Model<-"L"

SlopeLDIC$Model<-"L"

x <- W

y <- TotalSoftTissue

bdata <-bugsData(c("y","x"),,digits=5)

modelCheck(regressionmodelfile)

modelData(bdata)

modelCompile(numChains=1)

modelInits(inits,)

modelUpdate(50000)

samplesSet(c("b","R2B","a","prec"))

dicSet()

modelUpdate(50000)

SlopeW <- samplesStats("*")

SlopeWDIC <- dicStats()

SlopeW$Model<-"W"

SlopeWDIC$Model<-"W"

x <- H

y <- TotalSoftTissue

bdata <-bugsData(c("y","x"),,digits=5)

modelCheck(regressionmodelfile)

modelData(bdata)

modelCompile(numChains=1)

modelInits(inits,)

modelUpdate(50000)

samplesSet(c("b","R2B","a","prec"))

dicSet()

modelUpdate(50000)

SlopeH <- samplesStats("*")

SlopeHDIC <- dicStats()

SlopeH$Model<-"H"

SlopeHDIC$Model<-"H"

regressionmodel <- function(){

a~dgamma(0.001,0.001)

b~dnorm(0,1.0E-6)

prec~dgamma(0.001,0.001)

sy2 <- pow(sd(y[]),2)

R2B <- 1 - 1/(prec*sy2)

for (i in 1:50)

{

z[i]<- pow(x[i],b)

mean [i] <- a*z[i]

y[i] ~dnorm(mean[i],prec)

}

}

regressionmodelfile <- file.path(tempdir(),"regressionmodel.txt")

model <- writeModel(regressionmodel,regressionmodelfile)

inits <- "C:\\Users\\jhlarson\\Desktop\\USGS Science\\OpenBugs Code\\Regression\\InitialsAllo.txt"

x <- L

y <- TotalSoftTissue

bdata <-bugsData(c("y","x"),,digits=5)

modelCheck(regressionmodelfile)

modelData(bdata)

modelCompile(numChains=1)

modelInits(inits,)

modelUpdate(50000)

samplesSet(c("b","R2B","a","prec"))

dicSet()

modelUpdate(50000)

AlloL <- samplesStats("*")

AlloLDIC <- dicStats()

AlloL$Model<-"AlloL"

AlloLDIC$Model<-"AlloL"

x <- W

y <- TotalSoftTissue

bdata <-bugsData(c("y","x"),,digits=5)

modelCheck(regressionmodelfile)

modelData(bdata)

modelCompile(numChains=1)

modelInits(inits,)

modelUpdate(50000)

samplesSet(c("b","R2B","a","prec"))

dicSet()

modelUpdate(50000)

AlloW <- samplesStats("*")

AlloWDIC <- dicStats()

AlloW$Model<-"AlloW"

AlloWDIC$Model<-"AlloW"

x <- H

y <- TotalSoftTissue

bdata <-bugsData(c("y","x"),,digits=5)

modelCheck(regressionmodelfile)

modelData(bdata)

modelCompile(numChains=1)

modelInits(inits,)

modelUpdate(50000)

samplesSet(c("b","R2B","a","prec"))

dicSet()

modelUpdate(50000)

AlloH <- samplesStats("*")

AlloHDIC <- dicStats()

AlloH$Model<-"AlloH"

AlloHDIC$Model<-"AlloH"

SlopesTotalSoftTissue <-rbind(SlopeL,SlopeW,SlopeH,AlloW,AlloH,AlloL)

SlopesTotalSoftTissueDIC <-rbind(SlopeLDIC,SlopeWDIC,SlopeHDIC,AlloWDIC,AlloHDIC,AlloLDIC)

SlopesTotalSoftTissue$MC_error<- NULL

SlopesTotalSoftTissue$start<-NULL

SlopesTotalSoftTissue$sample<- NULL

SlopesTotalSoftTissue$Parameters <- SlopesTotalSoftTissue$row.names

SlopesTotalSoftTissueDIC<-SlopesTotalSoftTissueDIC[c(-2,-4,-6,-8,-10,-12),]

TotalSoftTissueResults<-join(SlopesTotalSoftTissue,SlopesTotalSoftTissueDIC,by="Model")

TotalSoftTissueResults$Parameters <- SlopesTotalSoftTissue$row.names
